# Anticancer active trifluoromethylated fused triazinones are safe for early-life stages of zebrafish (*Danio rerio*) and reveal a proapoptotic action

**DOI:** 10.1080/14756366.2020.1865944

**Published:** 2021-01-04

**Authors:** Małgorzata Sztanke, Jolanta Rzymowska, Krzysztof Sztanke

**Affiliations:** a Department of Medical Chemistry, Medical University, Lublin, Poland; b Department of Biology and Genetics, Medical University, Lublin, Poland; c Laboratory of Bioorganic Synthesis and Analysis, Department of Medical Chemistry, Medical University, Lublin, Poland

**Keywords:** Trifluoromethylated fused triazinones, *in vivo* zebrafish toxicity test, maximal non-lethal concentration, developmental defects, structure–toxicity relationships, caspase activation, proapoptotic action

## Abstract

The main purpose of this investigation was to evaluate the effect of anticancer active compounds (**I–VIII**) on zebrafish development in order to select the safest molecules. Larval mortality, embryo hatchability and malformations were end-points used to assess the acute toxicity among embryos and larvae from compounds-/pemetrexed-treated and control groups. LC_50_ and MNLC (maximal non-lethal concentration) were determined. Lipophilicity-dependent structure–toxicity relationships were established. The results clearly indicated that the majority of test molecules are safe for zebrafish individuals and simultaneously are less toxic than an anticancer agent – pemetrexed. The subsequent aim of this study was to elucidate the molecular mechanism of antiproliferative activity of the most selective compounds. Substantially increased activation of caspase-6 and -8 in cancerous cell lines confirmed the proapoptotic action of molecules examined. Considering the safety for zebrafish individuals, the title compounds as inducers of apoptosis are promising drug candidates in the preclinical phase of drug development.

## Introduction

**Figure 1.**
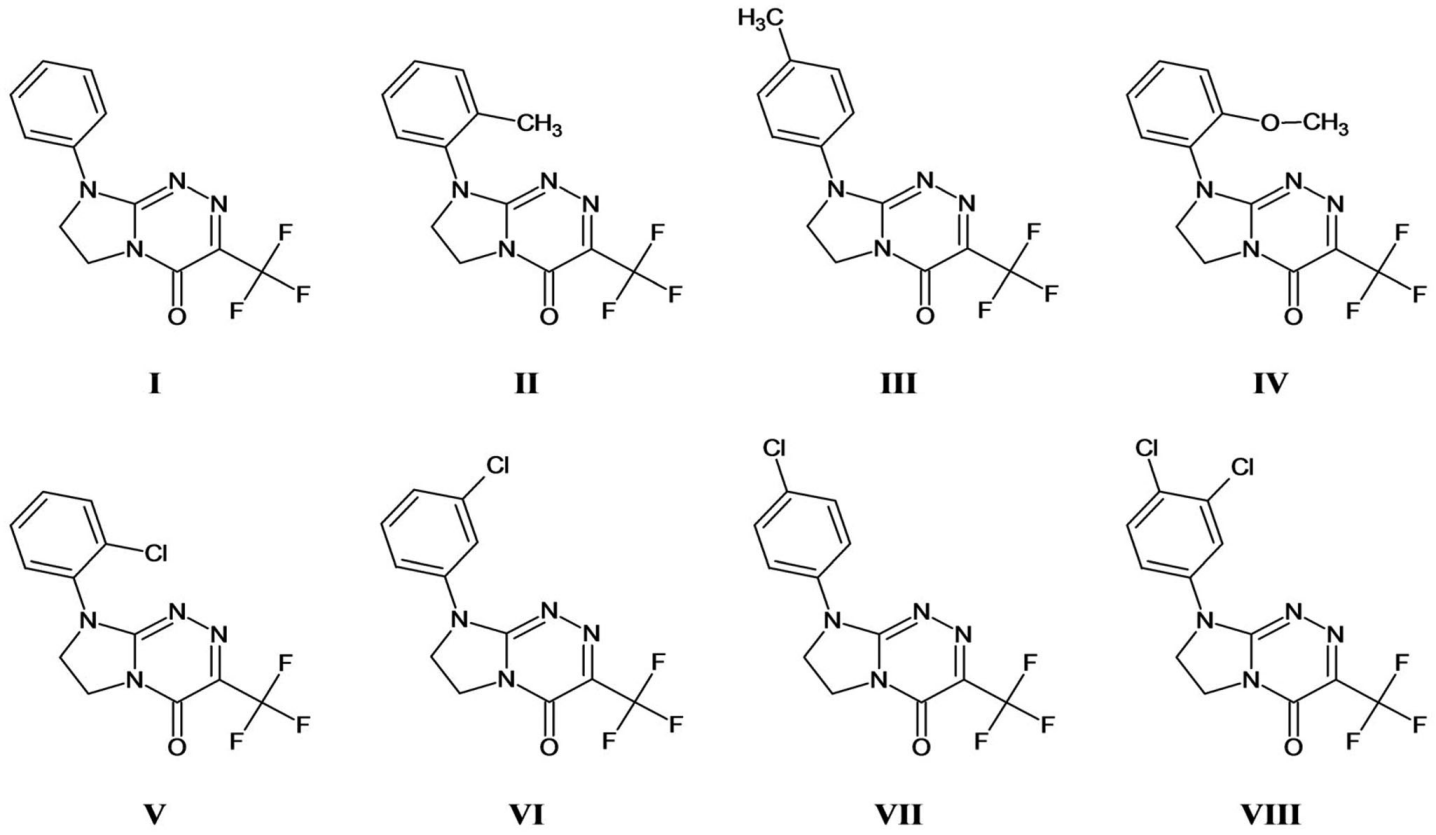
Structures of the studied molecules (**I–VIII**). Compounds are ordered in relation to the presence of different substituent attached at *N*8 of the privileged scaffold as follows: a parent structure, i.e. the phenyl derivative (**I**), *ortho*-methylphenyl derivative (**II**), *para*-methylphenyl derivative (**III**), *ortho*-methoxyphenyl derivative (**IV**), *ortho*-chlorophenyl derivative (**V**), *meta*-chlorophenyl derivative (**VI**), *para*-chlorophenyl derivative (**VII**), 3,4-dichlorophenyl derivative (**VIII**). All the investigated molecules (**I–VIII**) possess the common privileged 7,8-dihydroimidazo[2,1-*c*][1,2,4]triazin-4(6*H*)-one template and the trifluoromethyl group (showing strong electron-withdrawing effect) at *C*3.

Taking into account the widely highlighted significance of the trifluoromethyl group in pharmacologically active compounds and drugs^1–5^, the search for new non-toxic or low-toxic trifluoromethylated drug candidates is an important goal in current medicinal chemistry. Hence, we recently developed eight new 8-substituted-3-(trifluoromethyl)-7,8-dihydroimidazo[2,1-*c*][1,2,4]triazin-4(6*H*)-ones^6–8^ (**I–VIII**, Figure 1) – belonging to innovative antimetabolites that can act as suicide substrates (mechanism-based irreversible inactivators of specific enzymes). They revealed strong antiproliferative effects (which – in the future – may be useful in the treatment of human solid tumours of the breast, cervix and lung) as well as protective effects on erythrocytes exposed to some reactive oxygen species^6–8^. In addition, it has been observed that they show lipophilicity indices providing *inter alia* an optimal intestinal absorption, an effective permeability and a binding with albumin in human serum^8^. Most of the title trifluoromethylated molecules have been shown to possess stronger anticancer activities and better selectivity indices than that of pemetrexed (a clinically approved anticancer agent, belonging to the class of folate antimetabolites). In addition, three compounds of this novel class – containing the *ortho-*methyl*, ortho-*methoxy and *ortho-*chloro substituent at the phenyl moiety (**II**, **IV** and **V**, respectively) – proved to be the most selective, displaying *in vitro* less toxic effects to normal cells than pemetrexed^8^. Therefore, a whole set of these pharmacologically important and bioavailable molecules^6–8^ – with the anticipated resistance to metabolic deamination^8^ and bearing the valuable trifluoromethyl group (*inter alia* resistant to *in vivo* oxidation^9^^,^^10^) – seems to be the most appropriate for further *in vivo* studies.

The zebrafish (*Danio rerio*) is one of the well-known and popular *in vivo* animal models that are extensively used in toxicological and biomedical studies^11–19^. The high consistency between mammalian and zebrafish developmental toxicity was confirmed in a number of investigations^11^^,^^20^^,^^21^. Due to various advantages of zebrafish – such as the high level of homology to the human genome, structural similarities with vertebrates, the high reproductive ability, the optical transparency of embryos, very rapid external embryonic development, well-characterised behaviour, small size and inexpensiveness – this unique animal model can be an interesting tool for assessing the toxicity of new drug candidates^11^^,^^14^^,^^19^^,^^21–24^. This modern vertebrate model is popular in testing the safety of new drug-like compounds. The early-life stages of zebrafish, mainly embryos and non-feeding larvae, are considered more sensitive to chemical compounds than adult individuals and can therefore serve as an ideal screening tool in the preclinical phase of drug development or repositioning^11^^,^^14^^,^^16^^,^^23^^,^^25^. Zebrafish embryogenesis is completed within 72 h after fertilisation, while the major organs and tissues are developed within 120 h after fertilisation, making it easy to track drug-induced effects during different developmental stages^15^^,^^26^. Zebrafish can be a useful model for fast, reliable and low-cost studying the safety of new drug candidates^11^^,^^14^^,^^16^^,^^23^.

Due to the fact that the trifluoromethylated fused triazinones (**I–VIII**) showed significant pharmacological activities, selectivities as well as favourable pharmacokinetic properties, which have been recently patented^6^^,^^7^ and published^8^, they can be considered as valuable anticancer drug candidates in the preclinical phase of drug development. Therefore, it was necessary to extend studies on this class of molecules by assessing their toxicity profile in a modern vertebrate model as well as their proapoptotic action in caspase-based assays.

There are no scientific reports on the influence of the title fluorinated compounds on the embryonic and larval development of zebrafish. Only *in vitro* studies have been recently performed and their promising results have been published by our research group^6–8^. Therefore, the main scientific objective of the present study was evaluating the effects of promising trifluoromethylated compounds (**I–VIII**) on the early-life stages of zebrafish and selecting the safest compounds for further research. To accomplish this – for the first time – the mortality, hatching rate and developmental abnormalities among embryos and larvae from the compounds-treated groups were assessed, while the half maximal lethal concentration (LC_50_) and the maximal non-lethal concentration (MNLC) values for each test molecule were determined. Simultaneously, the influence of the title compounds on the embryonic and larval development of zebrafish was compared with the effect (in the same animal model system) of pemetrexed – a clinically used anticancer agent. Due to the fact that the investigated trifluoromethylated molecules differ in substituents at *N*8, the relationships between structure of the compounds and their toxicity for *Danio rerio* were determined. These explorations were undertaken in order to reveal the most favourable substitution patterns in test molecules, which would be safe for the normal development of zebrafish embryos/larvae. Moreover, the correlations between lipophilicity and toxicity of the compounds were established. The results of these pioneering studies on the title compounds, assessing for the first time their safety profile for vertebrates, are of the particular importance, novelty and originality. A toxicological characterisation of the compounds in this vertebrate model will be helpful in selecting the safest trifluoromethylated molecules for testing in mammals.

The subsequent purpose of our study was expanding the pharmacological research on the molecular mechanism of antiproliferative activity of the title compounds. Considering the importance of inducing apoptosis in the effectiveness of anticancer treatment^27^, further investigations were purposed at checking the effect of the most selective molecules (**II** and **V**) on the activation of caspase-5, -6 and -8. These proteases were chosen because of their function. Caspase-8 acts as an initiator of apoptosis, while caspase-6 is included in the group of executive proteins – playing a role in the final phase of apoptosis. In turn, caspase-5 reveals a pro-inflammatory function^27^.

## Materials and methods

### The investigated compounds (I–VIII)

8-Substituted-3-(trifluoromethyl)-7,8-dihydroimidazo[2,1-*c*][1,2,4]triazin-4(6*H*)-ones (**I–VIII**, Figure 1), which structures are supported by their consistent spectroscopic data, were resynthesised for the purposes of current research needs, according to synthetic procedures reported lately. The physico-chemical, structural, pharmacological and pharmacokinetic characterisation of these compounds has been recently published^6–8^.

### In vivo studies

#### Zebrafish embryo acute toxicity studies

The *in vivo* studies on toxicity of the investigated trifluoromethylated compounds (**I–VIII**) and pemetrexed towards zebrafish were conducted on the basis of Guidelines for the Testing of Chemicals of the Organisation for Economic Cooperation and Development (OECD)^28^. A modified zebrafish embryo acute toxicity test was carried out following the procedures described previously^29^.

#### Test compound solutions

To prepare each test solution, each pure compound in its solid state (**I–VIII**) was dissolved in dimethyl sulfoxide (DMSO; POCH SA, Gliwice, Poland) and then diluted with the E3 medium (i.e. a purified water containing 5 mM NaCl, 0.17 mM KCl, 0.33 mM CaCl_2_·H_2_O, 0.33 mM MgCl_2_·6 H_2_O and adjusted to pH 7.2) to obtain the appropriate concentration in the range of 1–100 mg L^−1^. Concentrations 1, 2.5, 5, 10, 25, 50 and 100 mg L^−1^ were selected on the basis of the initial screenings. DMSO – used as a solvent – had no embryotoxic effects on zebrafish at all the tested concentrations. Hence, there was no need to include a DMSO control group. In this experiment, an anticancer agent – pemetrexed (Sigma-Aldrich, Saint Louis, MO, USA) at the same concentrations as test compounds served as a positive control, whereas the E3 medium – as a negative control. All the solutions were always freshly prepared just prior to the experiment.

#### Embryo collection and exposure to test compounds

The zebrafish (*Danio rerio*) individuals came from the Experimental Medicine Centre, Medical University in Lublin, Poland. All the experiments on embryos and non-feeding larvae were carried out at this Experimental Medicine Centre.

As soon as possible after fertilisation, the eggs were transferred into Petri dishes (Sigma-Aldrich, Saint Louis, MO, USA) containing a fresh E3 medium. After washing and removing debris, only viable fertilised eggs with a round chorion were placed into 24-well sterile cell culture plates (Costar, Corning Inc., USA) (five embryos per well). Each well contained 2 ml of the test solution at a respective concentration or the E3 medium alone. Twenty embryos were used for each concentration of compound/pemetrexed. An equal number of embryos untreated with any compound/pemetrexed was employed as the control group. Throughout the experiment, all the covered plates with embryos/larvae were kept in the incubator at static conditions at 26 ± 1 °C up to 120 h after fertilisation.

#### Toxicity assessment of test compounds

The acute toxicity of the compounds (**I–VIII**) and pemetrexed on the embryonic/larval stages of zebrafish was assessed for a period of 5 days after fertilisation. Every 24 h, the mortality, hatchability and developmental malformations among individuals derived from both control and treated groups were observed using a stereomicroscope (Zeiss, SteREO Discovery.V8, Göttingen, Germany). The half maximal lethal concentration (LC_50_) and the maximal non-lethal concentration (MNLC) values of each compound as well as a standard drug were calculated based on the mortality at the end of a five-day exposure of the developing zebrafish to different concentrations of compounds/pemetrexed in three independent experiments with similar experimental conditions. The LC_50_ value (calculated by the probit method^30^) means the lowest concentration of compound/pemetrexed that causes death of half (50%) of zebrafish embryos/larvae during the duration of the experiment. The MNLC value represents the maximal concentration of compound/pemetrexed that does not result in a statistically different mortality of zebrafish embryos/larvae compared to the control group.

During the experiment, all zebrafish embryos/larvae that were identified as dead were removed, in order not to affect the surviving individuals.

#### Ethic statement

Experiments were performed in accordance with the European Union Directive^31^ as well as an applicable Polish legislation. The studies were conducted on the earliest life-stages of zebrafish (*Danio rerio*) which are not defined as protected. Therefore, their use for scientific purposes is not subject to regulations for animal experimentation.

### In vitro studies

#### Measurement of the effect of compounds II and V on caspase-5, -6 and -8 levels in normal and tumour cells

The caspase-5, -6 and -8 levels were evaluated employing Human Caspase-5, -6, -8 (CASP5, CASP6, CASP8) ELISA kits (based on the double-antibody sandwich enzyme-linked immunosorbent assay), which were purchased from SunRed Biotechnology Company (Shanghai, China). All the reagents, standards as well as cell culture supernatants were prepared according to the instructions that were given in the manufacturer kits. Lysates of normal (i.e. Vero) or tumour (i.e. A549, HeLa, T47D) cells were obtained as a result of the thermal shock repeated three times (from liquid nitrogen i.e. −186 °C to 37 °C). Both untreated (controls) and treated (with each test compound at a concentration close to the IC_50_, i.e. 50 mg L^−1^) cell lysates were used in the bioassay. These ones were added to the micro ELISA plate wells that were pre-coated with the Human CASP5 or 6 or 8 monoclonal antibodies. Next, the biotinylated detection antibodies specific for the individual caspases and the conjugate of streptavidin-horseradish peroxidase were added successively. The immune complex which was formed was incubated at 37 °C for 60 min. Each plate was washed five times with a washing buffer to remove the uncombined enzyme. After adding the chromogen A and B solutions, the incubation was still continued at 37 °C for 10 min in the darkness. Then, a stop solution was added in order to terminate the enzyme-substrate reaction. All the optical densities, being proportional to the concentration of particular caspases, were determined within 10 min on an ELISA reader (BIO-TEC Instruments, USA) at λ = 405 nm. The human caspase-5, -6 and -8 levels (in ng mL^−1^) were calculated employing the standard curves for the particular caspases.

### Statistical analysis

All the experiments were carried out in triplicate. The results were averaged and presented as the mean ± SD. Statistical analyses were performed employing Statistica 9.1.PL (StatSoft, Cracow, Poland). *p* Values <0.05 were considered statistically significant with a 95% confidence.

## Results and discussion

### The influence of test compounds (I–VIII) on zebrafish embryos/larvae

The zebrafish model – showing the high consistency in developmental toxicity studies with mammals^11^^,^^20^^,^^21^– seems to be an excellent research tool to determine the embryo(larva)toxicity of anticancer active 8-substituted-3-(trifluoromethyl)-7,8-dihydroimidazo[2,1-*c*][1,2,4]triazin-4(6*H*)-ones. Therefore, in the present study, we investigated – for the first time – the effect of compounds **I–VIII** and a standard anticancer drug – pemetrexed on the early-life stages (embryos and non-feeding larvae) of *Danio rerio* in the concentration- and exposure time-dependent manner. To assess the developmental toxicity among embryos and larvae from the compounds-/pemetrexed-treated and control groups, the end-points such as the mortality of larvae, hatching rate of embryos and developmental abnormalities observed at every 24 h post-fertilisation until five days after the exposure to the compounds/pemetrexed were included.

**Figure 2.**
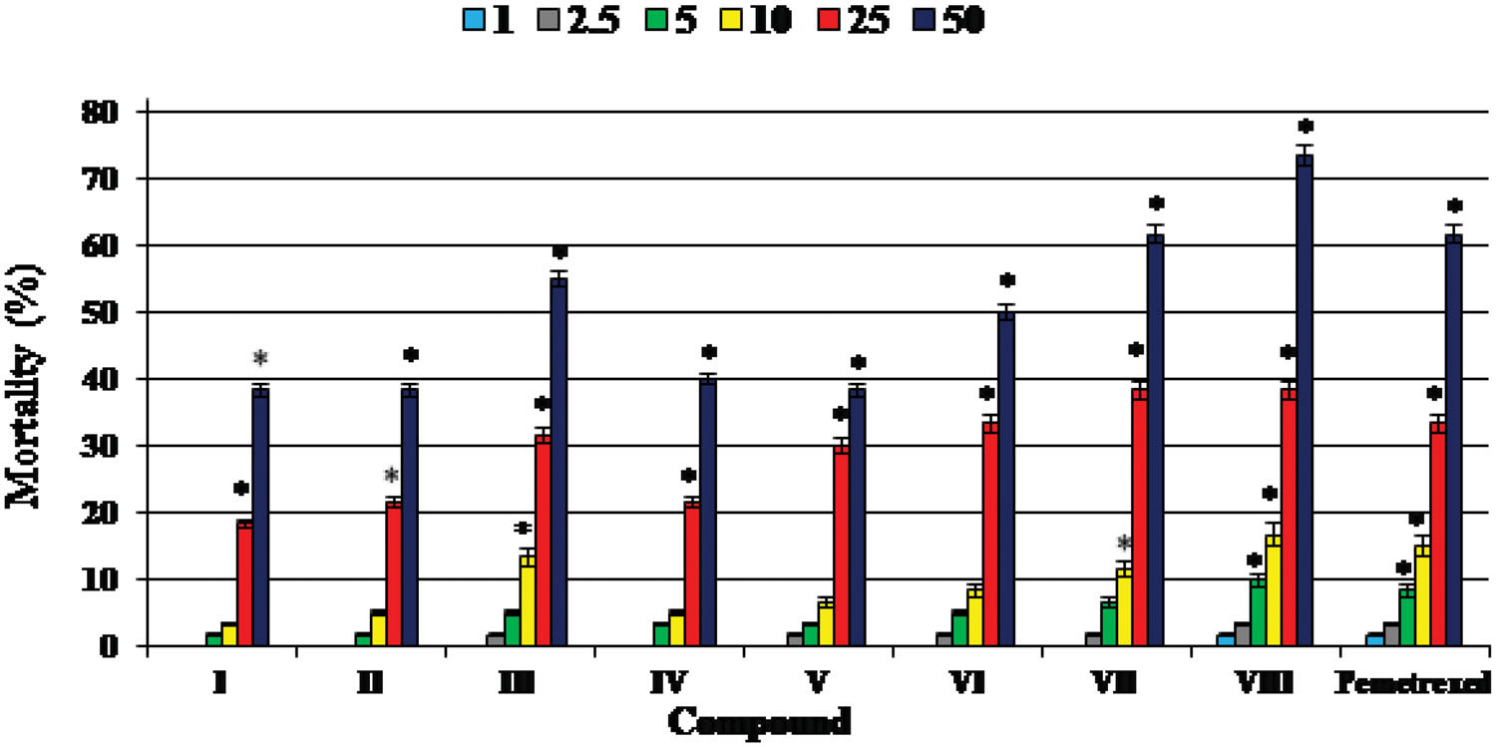
The mortality of zebrafish embryos/larvae at the end of five-day exposure to test compounds **I–VIII** and pemetrexed at concentrations of 1, 2.5, 5, 10, 25 and 50 mg L^−1^. The mortality in groups exposed to the compounds/pemetrexed at the concentration of 100 mg L^−1^ was 100%. The mortality rates were calculated taking the equation: (number of individuals that did not survive/n) × 100%. n – number of individuals exposed to a single dose of compound/pemetrexed in each experiment (n = 20). Data represent the mean ± SD of three independent experiments with similar experimental conditions. *Statistically significantly different from the control group (*p* < 0.05, Student’s *t*-test)

According to the OECD Guidelines for the Testing of Chemicals (Fish Embryo Acute Toxicity (FET) Test)^28^, the appropriate end-points for lethality are coagulation of the embryo, failure of somite development, non-detached tail and lack of heartbeat. The individuals showing at least one of these characteristics are considered dead. In our study, the indicator of mortality was the lack of heartbeat, and all the developing zebrafish identified as dead showed no heartbeat. We confirmed the death of embryos/larvae when their heartbeat was not observed under the microscope for at least one minute. The zebrafish mortality at the end of a five-day exposure to the trifluoromethylated fused triazinone (**I–VIII**) and pemetrexed at different concentrations was dose-dependent as shown in Figure 2.

It was found that the trifluoromethylated fused triazinones **I**, **II**, **IV**, **V** and **VI** at concentrations up to 10 mg L^−1^ (i.e. 1, 2.5, 5 and 10 mg L^−1^) and **III** and **VII** – at concentrations up to 5 mg L^−1^ (i.e. 1, 2.5 and 5 mg L^−1^) did not exhibit any significant mortality in zebrafish embryos and larvae at the end of five days of exposure to the compounds, as the mortality rates in these groups do not differ significantly from that in the control group (Figure 2). Whereas, at concentrations of 25 and 50 mg L^−1^ the mortality rates were elevated and were found be the highest for the compounds **III**, **VII** and **VIII** as well as pemetrexed. In turn, the highest concentration (100 mg L^−1^) of compounds/pemetrexed resulted in 100% mortality of zebrafish embryos and larvae during the experiment. Based on this, we calculated the lowest concentration that caused death of half of zebrafish embryos/larvae (LC_50_) for each test molecule as well as a standard anticancer drug (Table 1). It is noteworthy that the LC_50_ values of the vast majority of investigated compounds (i.e. **I–VI**) are higher than the LC_50_ value of pemetrexed, indicating that these trifluoromethylated molecules are safer for zebrafish embryos/larvae than this anticancer agent.

**Table 1.** The half maximal lethal concentration (LC_50_) and maximal non-lethal concentration (MNLC) values of the compounds **I–VIII** and pemetrexed for zebrafish embryos and larvae.

Compound	LC_50_ (95% CL^a^, mg L^−1^)^b^	MNLC (mg L^−1^)^b^,^c^
**I**	65 (53–76)	10 ± 0.0
**II**	68 (54–78)	15 ± 8.7
**III**	49 (40–62)	6.7 ± 2.9
**IV**	73 (62–84)	20 ± 8.7
**V**	64 (53–75)	10 ± 0.0
**VI**	56 (45–67)	8.3 ± 2.9
**VII**	38 (30–48)	5 ± 0.0
**VIII**	30 (22–40)	3.3 ± 1.4
Pemetrexed	40 (31–49)	4.2 ± 1.4

^a^ CL: confidence limit.

^b^ Data from three independent experiments with similar experimental conditions.

^c^ The mean ± SD.

**Figure 3.**
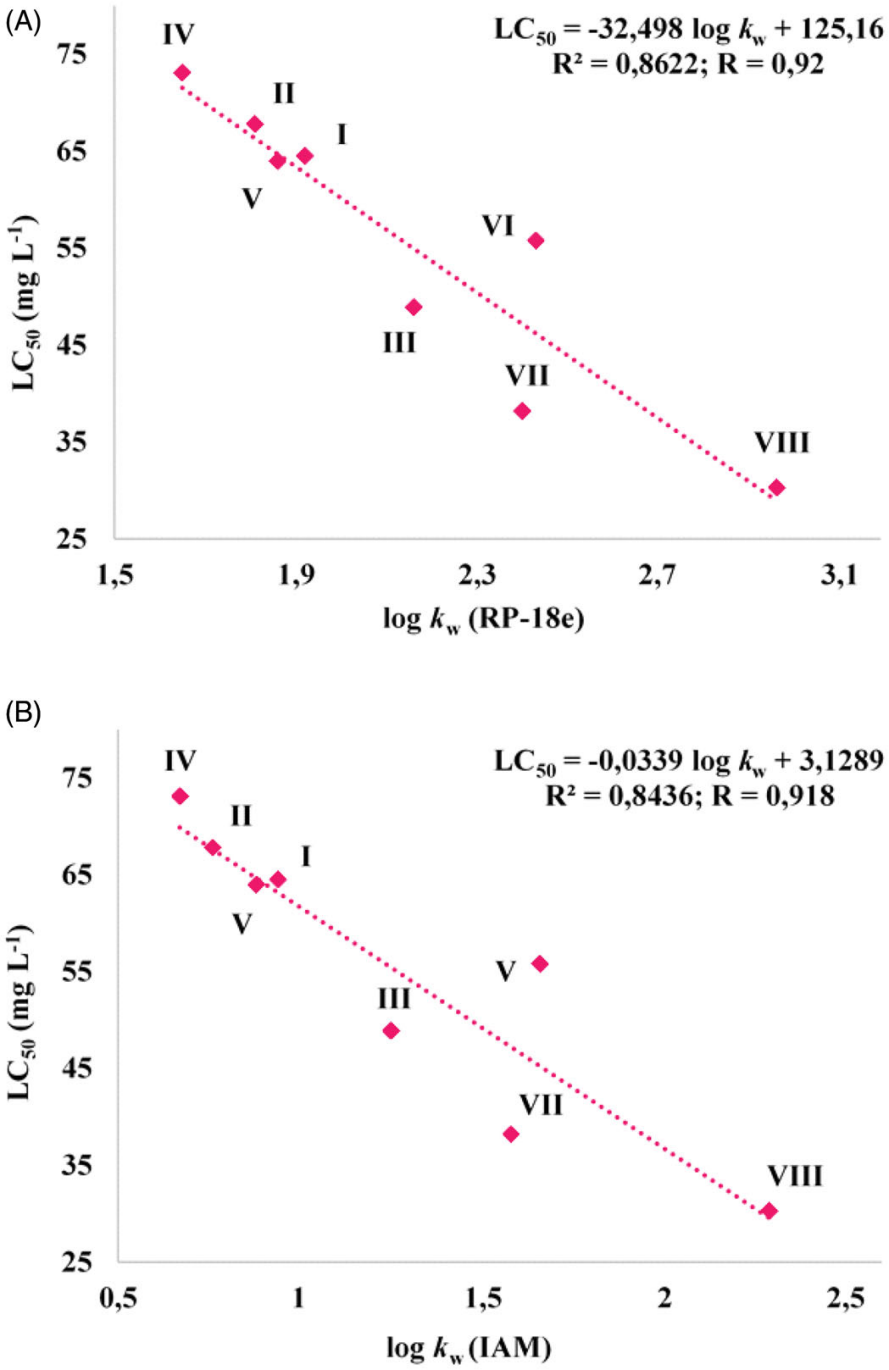
Correlations between LC_50_ values and lipophilicity indices of the trifluoromethylated fused triazinones (**I–VIII**) determined on an endcapped octadecylsilyl column (RP-18e) (A) and on an immobilised artificial membrane (IAM) phosphatidylcholine column (B). Log *k*_w_ (RP-18e) or (IAM) – the RP-HPLC capacity factor (in a logarithmic scale) of each compound in pure water as the mobile phase. All the standardized lipophilicity descriptors of **I-VIII** were established from their retention behaviour on the RP-18e or IAM column in water-acetonitrile buffered mobile phases.

Because the toxicity of chemical compounds may be dependent on their lipophilicity^10^, the correlations between previously determined lipophilicity indices^8^ and presented herein the LC_50_ values for test trifluoromethylated fused triazinones (**I–VIII**) were checked (Figure 3).

It was proven that the least lipophilic molecules (i.e. **IV**, **II**, **I**, **V**) and **VI** are the least toxic for zebrafish embryos/larvae, revealing the LC_50_ values from 73 to 56 mg L^−1^. Simultaneously, these compounds are less toxic than pemetrexed (LC_50_ = 40 mg L^−1^). In turn, it was noticed that the toxicity of more lipophilic compounds (**III** and **VII**) is similar to that of pemetrexed. However, it was established that only one compound with the highest experimental lipophilicity value (**VIII**) is slightly more toxic than pemetrexed. Our results are consistent with previous studies which showed that the toxicity of compounds and drug candidates to *Danio rerio* individuals usually increases with an increasing lipophilicity^32^^,^^33^. These data indicate that the exposure of zebrafish embryos and larvae to the trifluoromethylated fused triazinones **IV**, **II**, **I**, **V** and **VI** (bearing the *ortho*-methoxyphenyl, *ortho*-methylphenyl, phenyl, *ortho*-chlorophenyl and *meta*-chlorophenyl substitution, respectively) resulted in a lower mortality rate than the exposure to the anticancer drug – pemetrexed.

Additionally, the maximal non-lethal concentration (MNLC) – denoting the maximum concentration used which does not result in a statistically higher mortality rate of zebrafish embryos/larvae than in the control group during the experiment – for each examined trifluoromethylated fused triazinone (**I**–**VIII**) and pemetrexed was established (Table 1). It was proven that the molecules **IV**, **II**, **I**, **V** and **VI** (with the MNLC from 20 to 8.3 mg L^−1^) are less toxic than pemetrexed (with the MNLC = 4.2 mg L^−1^), while the toxicity of compounds **III** and **VII** (with the MNLC = 6.7 and 5 mg L^−1^, respectively) is close to that of this anticancer drug. Among all the compounds tested, only **VIII** (with MNLC = 3.3 mg L^−1^) was found to be slightly more toxic than pemetrexed.

Hatching – the transformation of an embryo to a larva – is a critical stage for embryonic development of aquatic organisms^14^^,^^15^^,^^23^. Normal zebrafish embryos hatch between 48 and 72 h post-fertilisation, and the ability to hatch may be stimulated or inhibited by various factors, including molecular pharmaceuticals^14^^,^^34^. Therefore, the hatchability of zebrafish is one of important parameters for assessing the toxicity of potential drug candidates^15^^,^^20^. The hatching rates of zebrafish embryos, exposed to each test trifluoromethylated fused triazinone (**I–VIII**) and pemetrexed at different concentrations, are presented in Figure S1 in Supplementary material. It was shown that the hatchability of embryos is not significantly affected by the compounds **I–VIII** or pemetrexed at concentrations of 1, 2.5, 5 and 10 mg L^−1^ when compared to the control group. Almost all the embryos from both untreated and treated groups hatched between 48 and 72 h post-fertilisation. However, the hatching rates were delayed in the compounds/pemetrexed groups exposed to 25 and 50 mg L^−1^ concentrations. Interestingly, even at the highest concentration, all living embryos were hatched out from the chorion at 96 h post-fertilisation (in case of **I**, **II**, **IV**, **V** and **VI**) or 120 h post-fertilisation (in case of **III**). In turn, a significant inhibition of hatching was observed in the embryos treated with the trifluoromethylated fused triazinones substituted by the 3,4-dichlorophenyl and *para*-chlorophenyl at *N*8 (i.e. **VIII** and **VII**, respectively) as well as pemetrexed. At the highest concentration almost all the embryos exposed to **VII**, **VIII** and pemetrexed were unable to hatch up to 120 h post-fertilisation.

**Figure 4.**
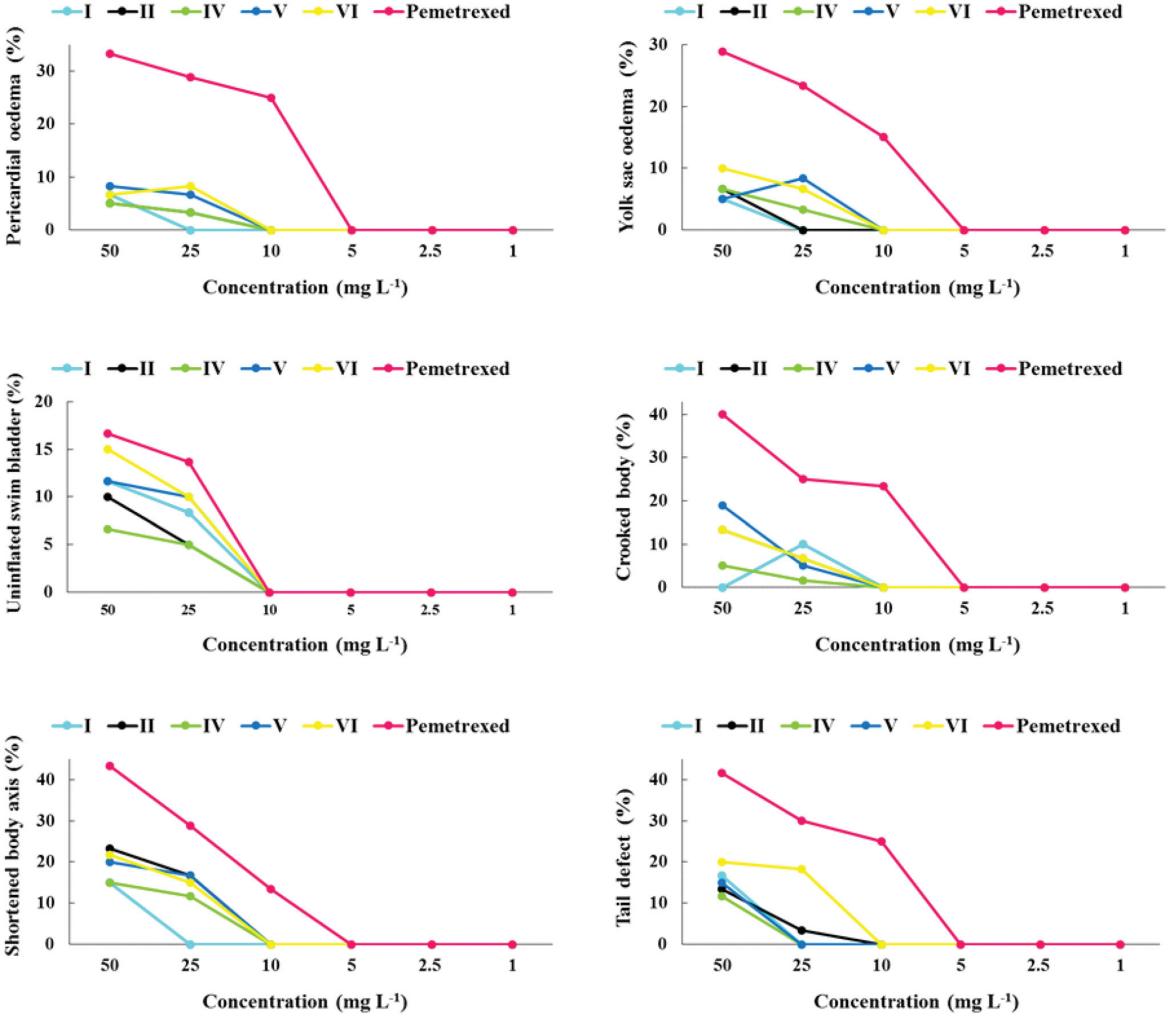
Types of developmental malformations in the early-life stages of zebrafish induced by compounds **I**, **II**, **IV**, **V** and **VI** (at different concentrations) that are safer than pemetrexed.

Developmental defects of zebrafish embryos and larvae are another very important indicators of a potential drug toxicity. Therefore, in testing the safety of drug candidates, the embryonic/larval developmental stages of zebrafish are carefully observed for potential deformities, such as the pericardial oedema, yolk sac oedema, uninflated swim bladder, crooked body, abnormal pigmentation, head and tail defects^15^^,^^16^^,^^26^^,^^35^. The effects of the investigated trifluoromethylated fused triazinones (**I–VIII**) and pemetrexed at 1–50 mg L^−1^ concentrations on the developing zebrafish at 24, 48, 72, 96 and 120 h after fertilisation are presented in Figures S2–S6 in Supplementary material. Types of developmental malformations, observed during the five-day exposure of zebrafish embryos and larvae to the compounds/pemetrexed, were summarised in Figure 4 and Figure S7 in Supplementary material, whereas the effects of test compounds/pemetrexed at the highest concentrations at different time points are shown in Figure S8 in Supplementary material.

**Figure 5.**
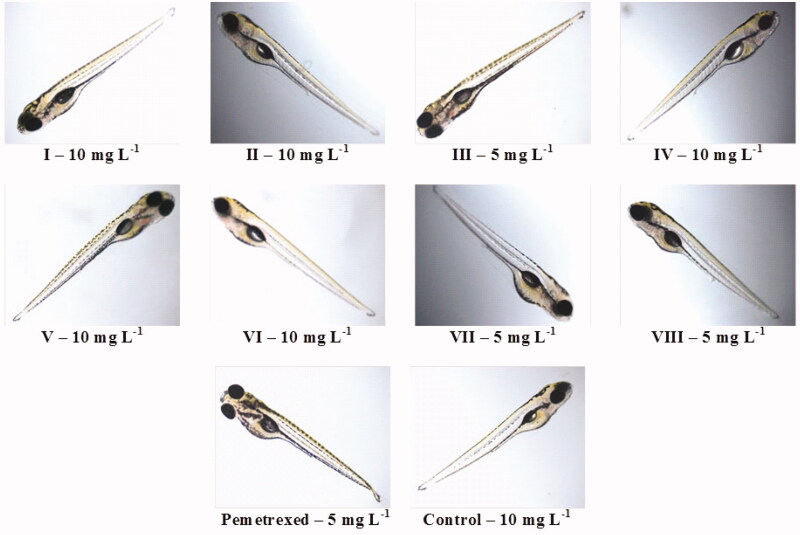
Zebrafish larvae at the end of five days exposure to the highest concentration of compounds (**I–VIII**) or pemetrexed that generally did not induce developmental defects.

At concentrations of 1–10 mg L^−1^ (in case of compounds **I**, **II**, **IV**, **V** and **VI**) (Figure 4), 1–5 mg L^−1^ (in case of **III**, **VII** and pemetrexed) and 1–2.5 mg L^−1^ (in case of **VIII**) (Figure S7 in Supplementary material), any significant developmental malformations were observed in the compounds-/pemetrexed-treated embryos/larvae when compared to the untreated control group. These *Danio rerio* individuals had normal embryonic development during the five-day exposure to the compounds/pemetrexed. In turn, the investigated trifluoromethylated fused triazinones and a standard anticancer drug at higher concentrations caused weak to severe deformities which were the exposure time- and chemical structure-dependent. The most severe malformation types were the accumulation of pellucid fluid in yolk sac as well as pericardium and failure of swim bladder inflation, which indicate that these organs (yolk sac, heart and swim bladder) may be sensitive to test compounds. However, it should be noted that these abnormalities were observed only in the developing zebrafish exposed to the highest compounds/pemetrexed concentrations. In turn, deformities such as the shortened body axis, tail defect and spinal curvature (suggesting gastrulation defects) were the most frequently observed in treated *Danio rerio* larvae. It is worth noting that the exposure of zebrafish embryos/larvae to the compounds **I**, **II**, **IV**, **V** and **VI** at each concentration tested (i.e. 1, 2.5, 5, 10, 25 and 50 mg L^−1^) resulted in less frequent and less severe developmental abnormalities than an exposure to the clinically useful pemetrexed (Figure 4). In contrast, the compounds **III**, **VII** and **VIII** showed a higher level of toxicity, as they induced intensified and more serious phenotypic defects in the developing *Danio rerio*, although these morphological changes were comparable to those caused by pemetrexed (Figure S7 in Supplementary material). Figure 5 shows zebrafish larvae at the end of five days of exposure to the highest concentration of compounds/pemetrexed that generally did not induce developmental abnormalities.

### The effect of compounds II and V on caspase-5, -6 and -8 activation in normal and tumour cells

Caspases are a class of cysteine-aspartic proteases with an important function in controlling cell death and inflammation. These enzymes play a crucial role in both initiation and execution of the programmed cell death – apoptosis. As apoptosis is mediated by caspases, it is important to search for activators of these proteases as potential effective anticancer agents^27^^,^^36^.

Our previous study^6–8^ confirmed the anticancer activity of 8-substituted-3-(trifluoromethyl)-7,8-dihydroimidazo[2,1-*c*][1,2,4]triazin-4(6*H*)-ones (**I–VIII**). These original molecules showed strong antiproliferative effects against cancerous (A549, HeLa and T47D) cell lines, while having less toxicity to the non-tumoural (Vero) cell line. A lower toxicity towards normal cells was due to the presence of the trifluoromethyl moiety. It was confirmed in comparative studies with their structural analogues with comparable steric rearrangement and bearing the ethyl or isopropyl groups^8^. In addition, most of the trifluoromethylated compounds were more effective at inhibiting the proliferation in tumour cells than pemetrexed (an anticancer drug), making them promising drug candidates^6–8^.

To investigate whether cell death proceeds through an apoptotic mechanism, the most selective trifluoromethylated compounds (bearing the *ortho*-methylphenyl (**II)** and *ortho*-chlorophenyl (**V**) at *N*8) were tested for their ability to activate some caspases in tumour cells of the lung (A549), cervix (HeLa), breast (T47D) as well as in normal (Vero) cells. The levels of caspase-8 (an initiator caspase) and -6 (an executioner caspase) – as apoptotic caspases – as well as caspase-5 (an inflammatory caspase) were measured.

**Figure 6.**
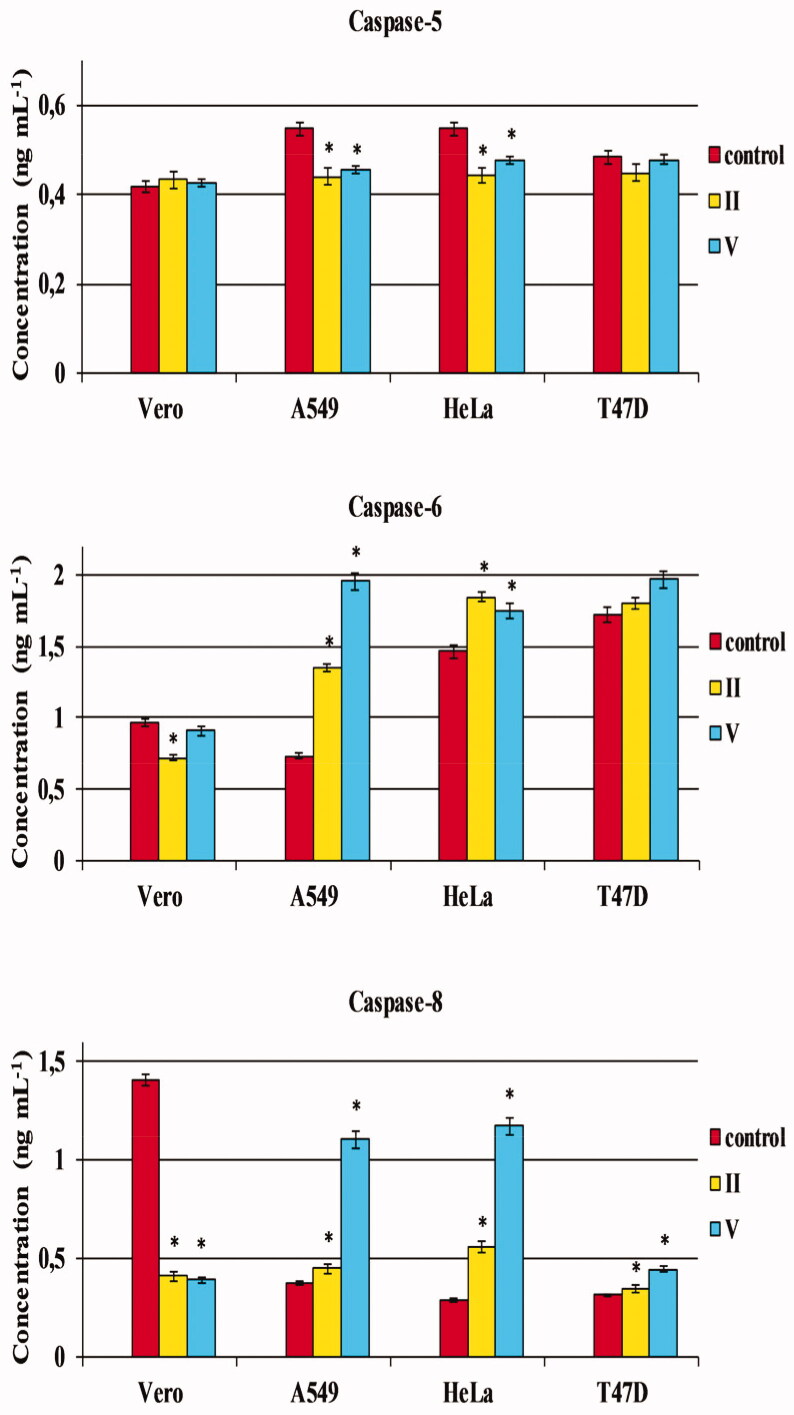
The effect of compounds **II** and **V** on caspase-5, -6 and -8 activation in normal and tumour cells. Normal cell line: Vero – (ECACC 88020401) – African Green Monkey kidney cells. Cancer cell lines: A549 (ECACC 86012804) – human Caucasian lung carcinoma cells; HeLa (ECACC 93021013) – human Negroid cervix epitheloid carcinoma cells; T-47D (ECACC 85102201) – human breast carcinoma cells. Data represent the mean ± SD of three independent experiments. *Statistically significantly different from the control (*p* < 0.05, *t*-Student’s test).

It was proven that the investigated molecules inhibit the activation of caspase-6 and -8 in normal Vero cells relative to the control. In turn, an activation of caspase-8 after incubation with test compounds was significantly enhanced in all the recruited cancerous cells (A549, HeLa and T47D), and a remarkable increase in caspase-6 level was observed in tumour cells of the lung (A549) and cervix (HeLa). The highest differences were observed for the trifluoromethylated molecule substituted by an *ortho*-chlorophenyl (**V)** (Table 2, Figure 6). These results indicate that the cytotoxic effects of test compounds may be attributed to the activation of caspases being the inducers of both initiation and executive phases of apoptosis. Moreover, a reduced level of caspase-5 was noticed in tumour A549 and HeLa cells after incubation with compounds **II** and **V**.

**Table 2.** The effect of compounds **II** and **V** on caspase-5, -6 and -8 activation in normal and tumour cells.

Cell line	Compound	Concentration (ng mL^−1^)		
		Caspase-5	Caspase-6	Caspase-8
Vero	Control	0.416 ± 0.02	0.962 ± 0.06	1.404 ± 0.5
	**II**	0.432 ± 0.02	0.720 ± 0.08*	0.408 ± 0.02*
	**V**	0.426 ± 0.01	0.906 ± 0.06	0.390 ± 0.05*
A549	Control	0.546 ± 0.06	0.732 ± 0.1	0.372 ± 0.1
	**II**	0.440 ± 0.05*	1.348 ± 0.8*	0.447 ± 0.2*
	**V**	0.454 ± 0.04*	1.958 ± 0.7*	1.105 ± 0.8*
HeLa	Control	0.546 ± 0.06	1.464 ± 0.6	0.286 ± 0.06
	**II**	0.442 ± 0.04*	1.846 ± 0.7*	0.554 ± 0.04*
	**V**	0.476 ± 0.04*	1.748 ± 0.8*	1.170 ± 0.8*
T47D	Control	0.484 ± 0.03	1.726 ± 0.5	0.312 ± 0.07
	**II**	0.448 ± 0.02	1.798 ± 0.7	0.346 ± 0.06*
	**V**	0.478 ± 0.03	1.970 ± 0.6	0.445 ± 0.08*

Normal cell line: Vero – (ECACC 88020401) – African Green Monkey kidney cells.

Cancer cell lines: A549 (ECACC 86012804) – human Caucasian lung carcinoma cells; HeLa (ECACC 93021013) – human Negroid cervix epitheloid carcinoma cells; T47D (ECACC 85102201) – human breast carcinoma cells.

Data represent the mean ± SD of three independent experiments.

*Statistically significantly different from the control (*p* < 0.05, *t*-Student’s test).

Summarising, the tested trifluoromethylated fused triazinones, due to the activation of caspases in cancer cells, can be considered as valuable inducers of apoptosis.

## Conclusions

The results of the present study clearly indicate that the vast majority of trifluoromethylated fused triazinones (*i. e.* **IV**, **II**, **I**, **V** and **VI)** are safe for the embryonic/larval developmental stages of *Danio rerio*. It should be noted that the five-day exposure of zebrafish embryos/larvae to most of test molecules (i.e. **I–VII**) at concentrations up to 10 or 5 mg L^−1^ had no significant effects on any of the parameters assessed. Simultaneously, the calculated maximal non-lethal as well as half maximal lethal concentrations for the compounds **IV**, **II**, **I**, **V** and **VI** are higher than that of an anticancer agent – pemetrexed, making them promising drug candidates in the preclinical phase of drug development. Noteworthy is that an increase in the embryo(larva)toxicity was observed in groups treated with compounds only at higher concentrations, and this toxicity was similar to that induced by pemetrexed. Among this class of small molecules, the trifluoromethylated fused triazinone structures **IV**, **II**, **I**, **V** and **VI** (i.e. bearing the *ortho*-chlorophenyl, *ortho*-methylphenyl, phenyl, *ortho*-methoxyphenyl and *meta*-chlorophenyl substitution, respectively) proved to be the most safe – and simultaneously less toxic than pemetrexed – for zebrafish embryos/larvae. Therefore, these molecules – with the most favourable substitution patterns affecting their less lipophilicity and therefore less toxicity *in vivo* – seem to be safe drug candidates based on their comparative studies with pemetrexed. Hence, they will be selected for further *in vivo* studies in mammals. From the point of view of medicinal chemistry, it is a significant finding in the search and development of new low-toxic drug candidates.

Moreover, the activation of caspase-6 and -8 (as inducers of programmed cell death) in tumour epithelial cell lines confirms the expected – *via* apoptosis – mechanism of action of the investigated compounds. Both 8–(2-methylphenyl)-3-(trifluoromethyl)-7,8-dihydroimidazo[2,1-*c*][1,2,4]triazin-4(6*H*)-one (**II**) and 8–(2-chlorophenyl)-3-(trifluoromethyl)-7,8-dihydroimidazo[2,1-*c*][1,2,4]triazin-4(6*H*)-one (**V**) – distinctly enhancing the activation of the above-mentioned caspases – can be considered as the selective apoptosis inducers. Considering the significance of apoptosis induction in the efficacy of anticancer therapy, the tested trifluoromethylated compounds, which proved to be caspases’ activators, seem to have the potential for future application in the treatment of human cancers of the lung, cervix and breast.

The results of *in vivo* zebrafish-based and *in vitro* biochemical studies, concerning the title trifluoromethylated fused triazinones, constitute a significant contribution to the current state of knowledge on preclinical toxicology and pharmacology of this novel class of compounds. These promising anticancer active and safe small molecules can be considered as suitable drug candidates for further more advanced investigations.

## Supplementary Material

Supplemental Material
