# Supplementary material for: Anticancer active trifluoromethylated fused triazinones are safe for early-life stages of zebrafish (Danio rerio) and reveal a proapoptotic action
Source: J Enzyme Inhib Med Chem. 2021 Jan 4;36(1):336–44. doi: 10.1080/14756366.2020.1865944 (PMC7782186; doi:10.1080/14756366.2020.1865944)
Supplement: Supplemental Material [file IENZ_A_1865944_SM4319.pdf]

Supplementary data

Anticancer active trifluoromethylated fused triazinones are safe for early-life stages of zebrafish (*Danio rerio*) and reveal a proapoptotic action

Małgorzata Sztanke[a]*, Jolanta Rzymowska[b], Krzysztof Sztanke[c]*

[a]Chair and Department of Medical Chemistry, Medical University, 4A Chodźki Street, 20-093 Lublin, Poland;
[b]Department of Biology and Genetics, Medical University, 4A Chodźki Street, 20-093 Lublin, Poland;
[c]Laboratory of Bioorganic Synthesis and Analysis, Chair and Department of Medical Chemistry, Medical University, 4A Chodźki Street, 20-093 Lublin, Poland

**Figure S1.** The hatching rates of zebrafish embryos exposed to test compounds (**I-VIII**) and pemetrexed at different concentrations (1, 2.5, 5, 10, 25 and 50 mg L^{-1})…………………………………………………	**2**
**Figure S2.** Zebrafish individuals from compounds- and pemetrexed-treated groups as well as from the control group (untreated) at a 24 hpf……………………..	**3**
**Figure S3.** Zebrafish individuals from compounds- and pemetrexed-treated groups as well as from the control group (untreated) at a 48 hpf……………………...	**4**
**Figure S4.** Zebrafish individuals from compounds- and pemetrexed-treated groups as well as from the control group (untreated) at a 72 hpf……………………...	**5**
**Figure S5.** Zebrafish individuals from compounds- and pemetrexed-treated groups as well as from the control group (untreated) at a 96 hpf……………………..	**6**
**Figure S6.** Zebrafish individuals from compounds- and pemetrexed-treated groups as well as from the control group (untreated) at a 120 hpf……………………..	**7**
**Figure S7.** Types of developmental malformations in zebrafish individuals induced by compounds **III, VII, VIII** and pemetrexed at different concentrations at a 120 h after fertilization……………………………........	**8**
**Figure S8.** Zebrafish individuals in an untreated control group (**A-E**) as well as in groups exposed to the highest concentrations of some test compounds (**F-J**) or pemetrexed (**K-O**)………………………………..	**9**

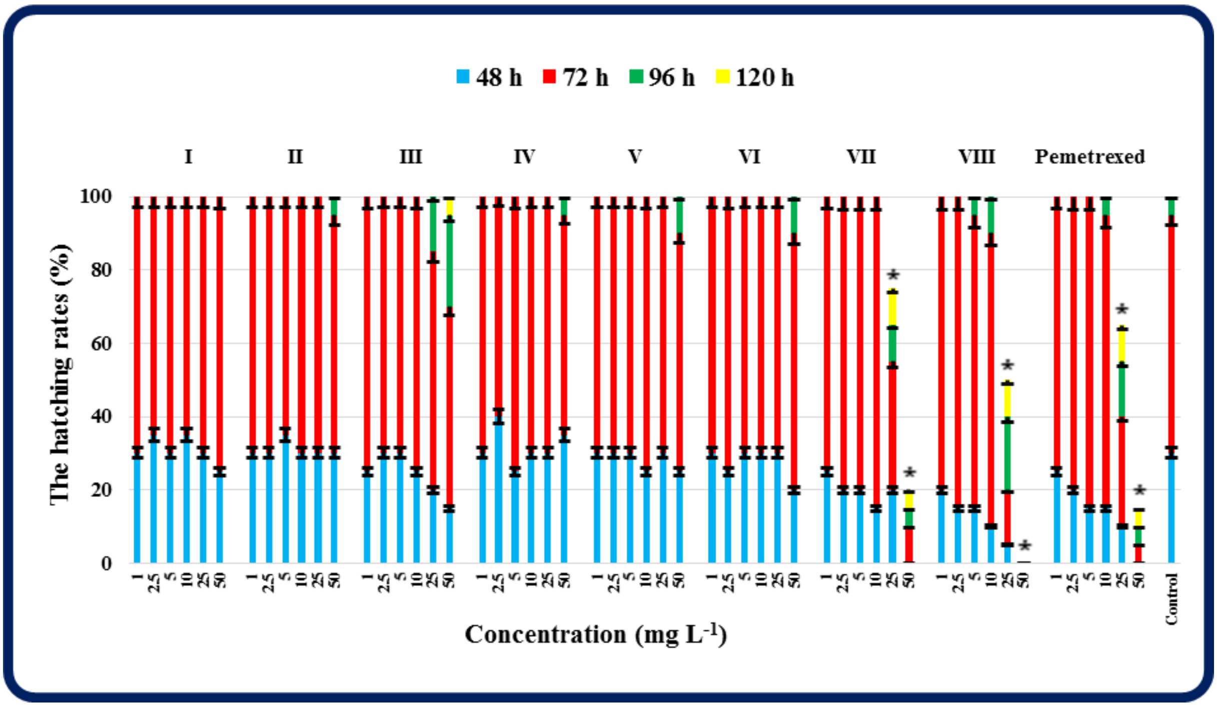

Figure S1. The hatching rates of zebrafish embryos exposed to test compounds (**I-VIII**) and pemetrexed at different concentrations (1, 2.5, 5, 10, 25 and 50 mg L^{-1}).

The data are not shown for compounds and pemetrexed at a concentration of 100 mg L^{-1} due to the lack of surviving embryos.

The hatching rates were calculated by using the equation: (number of hatched individuals / number of all survived individuals) x 100%.

Data represent the mean ± SD of three independent experiments.

* – statistically significantly different from the control group ($p < 0.05$, Student's *t*-test)

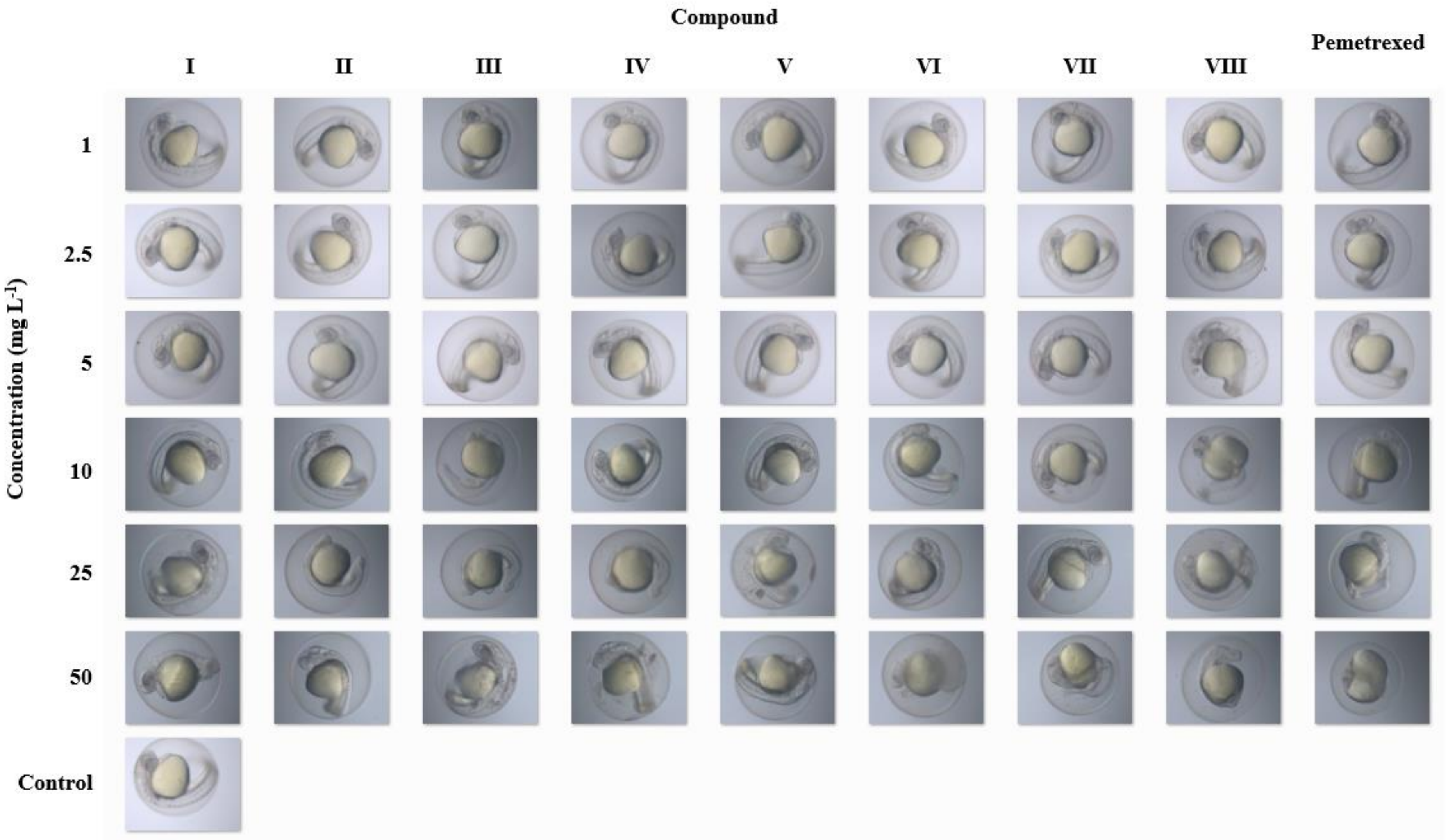

Figure S2. Zebrafish individuals from compounds- and pemetrexed-treated groups as well as from the control group (untreated) at a 24 hpf.

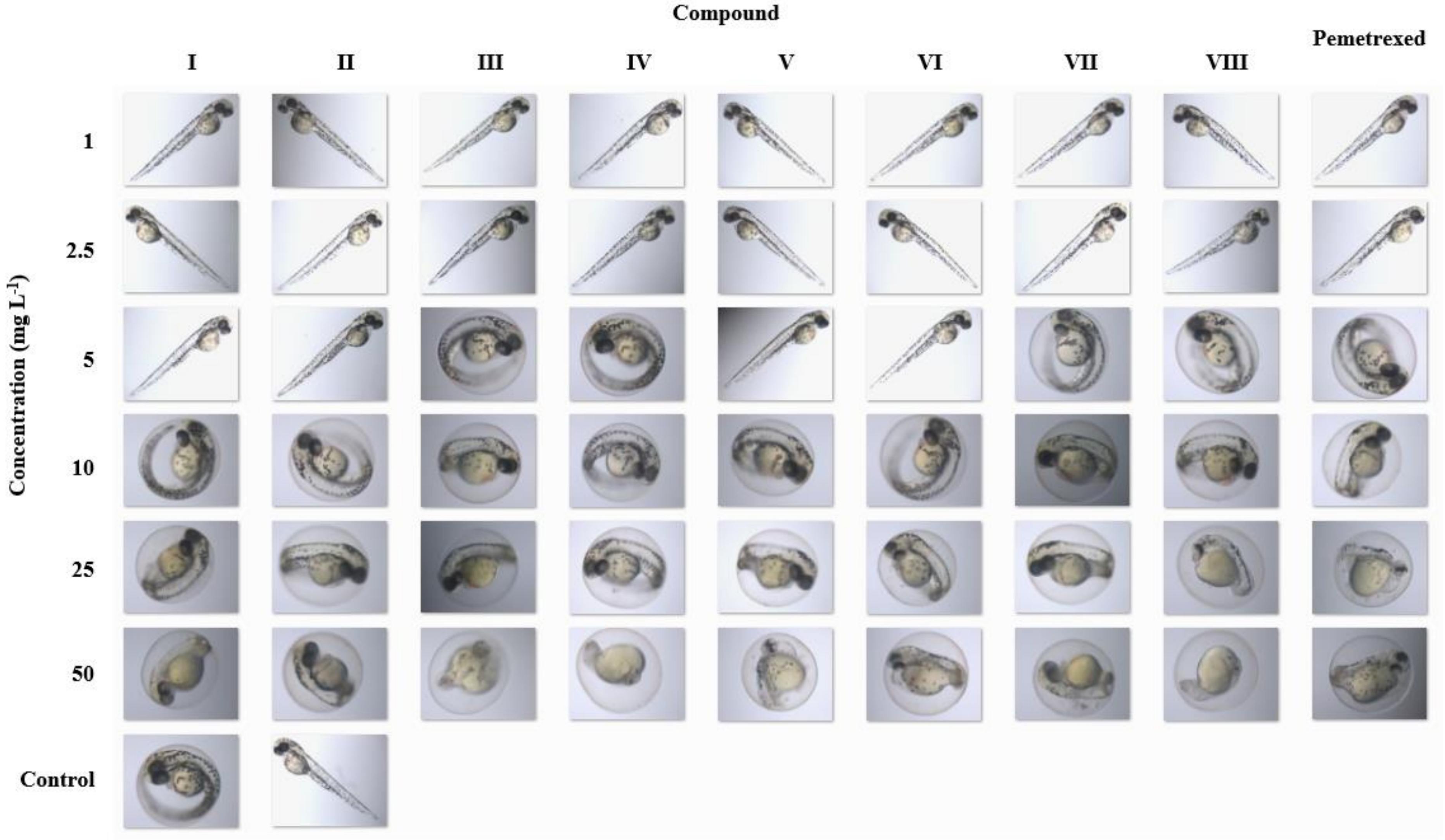

Figure S3. Zebrafish individuals from compounds- and pemetrexed-treated groups as well as from the control group (untreated) at a 48 hpf.

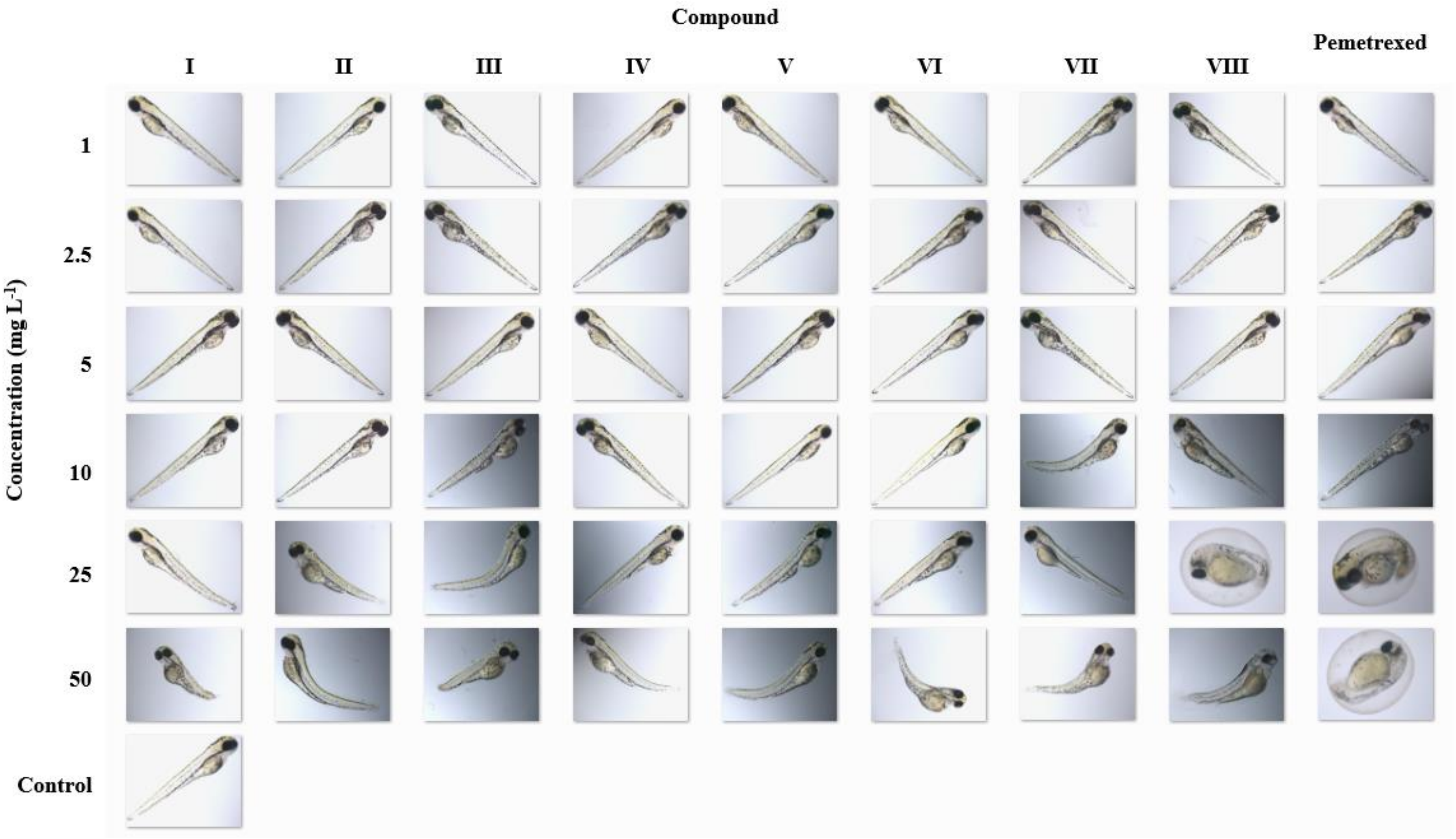

Figure S4. Zebrafish individuals from compounds- and pemetrexed-treated groups as well as from the control group (untreated) at a 72 hpf.

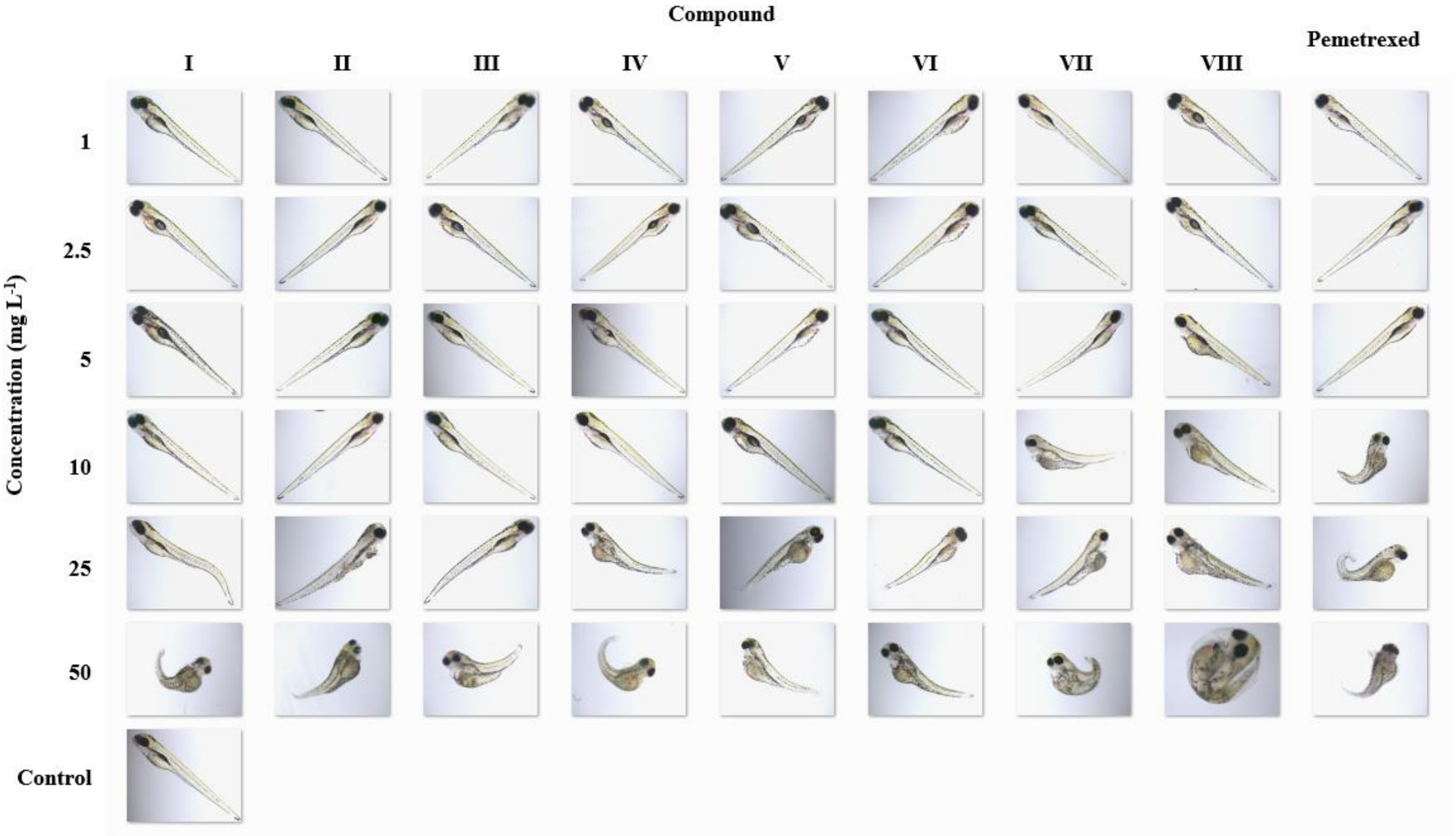

Figure S5. Zebrafish individuals from compounds- and pemetrexed-treated groups as well as from the control group (untreated) at a 96 hpf.

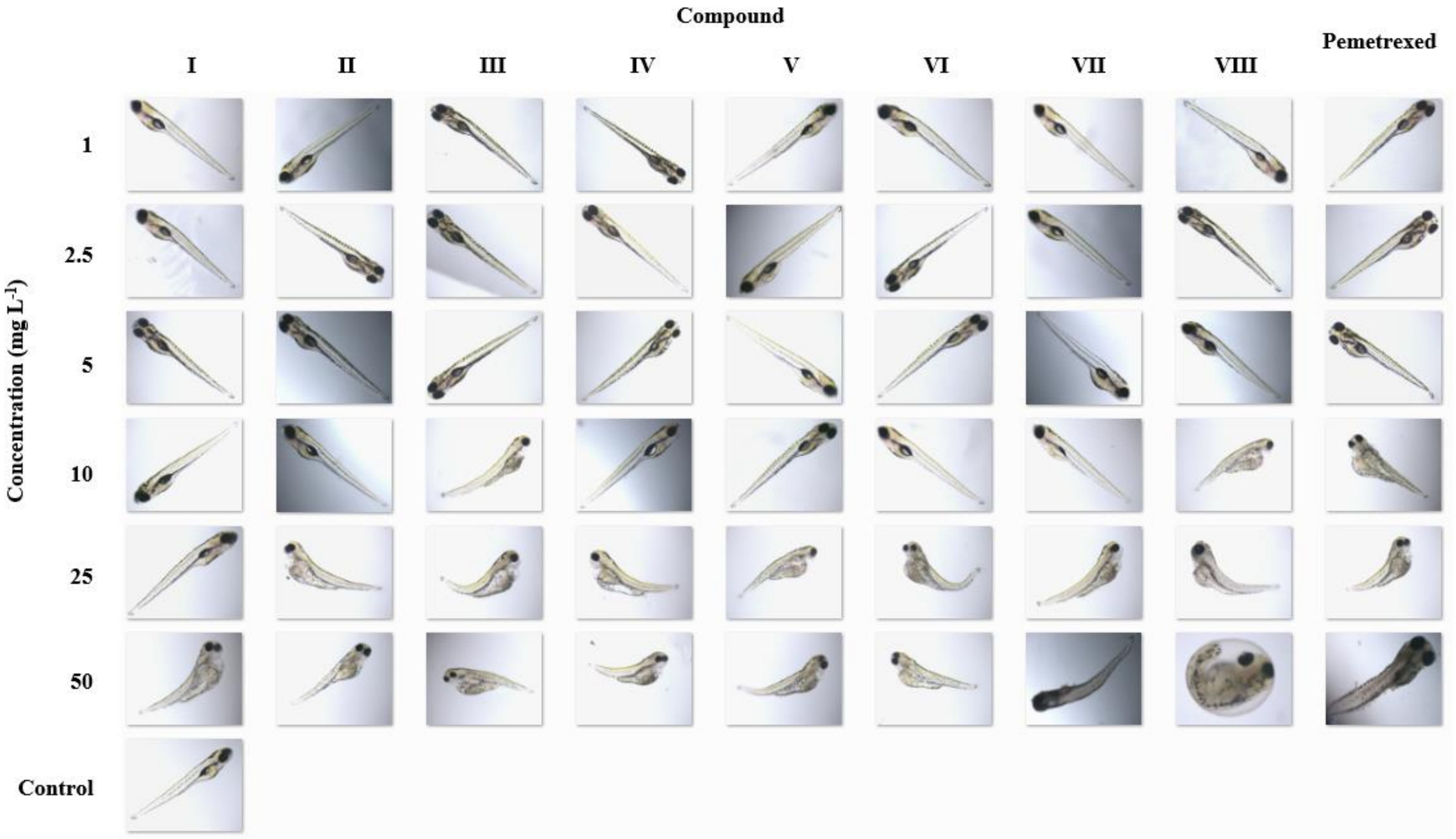

Figure S6. Zebrafish individuals from compounds- and pemetrexed-treated groups as well as from the control group (untreated) at a 120 hpf.

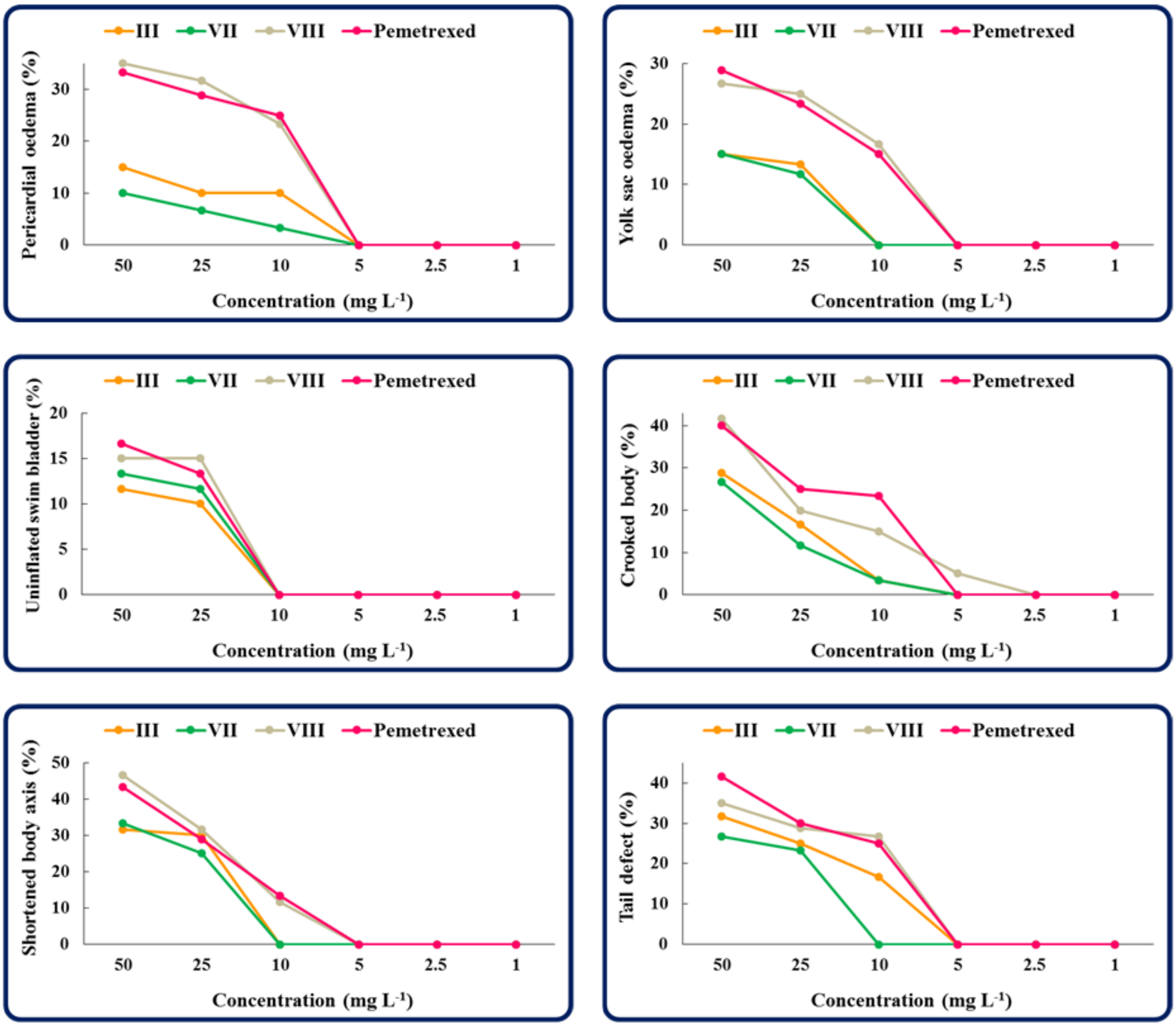

Figure S7. Types of developmental malformations in zebrafish individuals induced by compounds **III, VII, VIII** and pemetrexed at different concentrations.

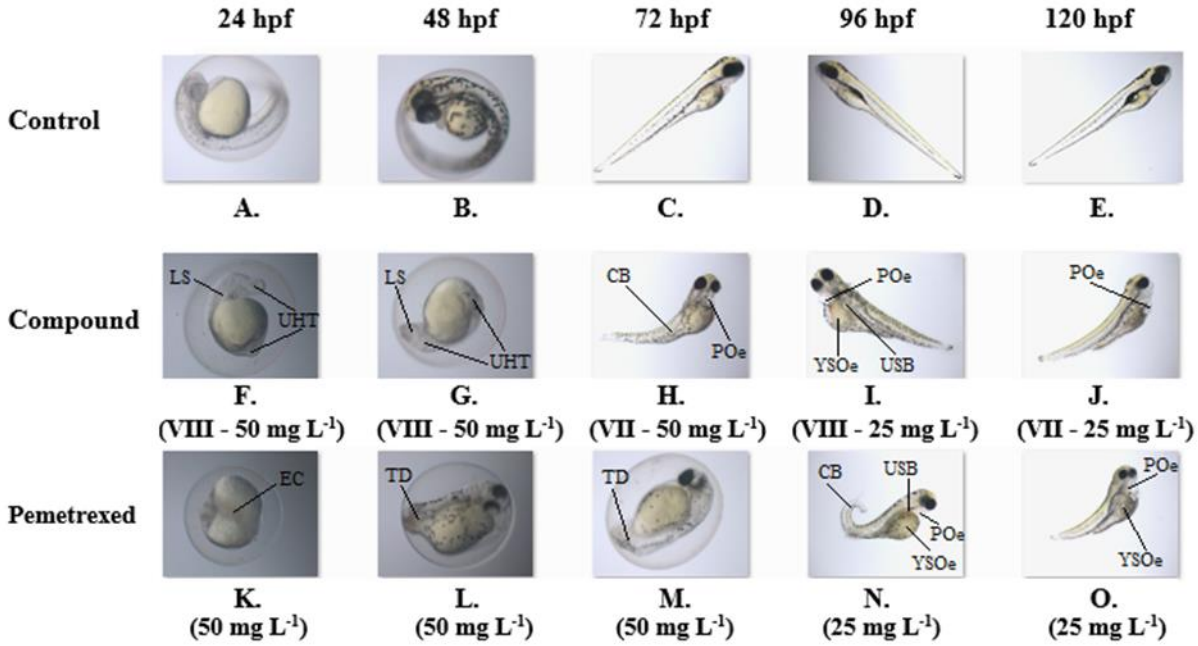

Figure S8. Zebrafish individuals in an untreated control group (**A-E**) as well as in groups exposed to the highest concentrations of some test compounds (**F-J**) or pemetrexed (**K-O**). Embryos in a control group with well-developed head and tail and clearly visible somites (**A-B**). Larvae in a control group with the normal body structure, well-developed craniofacial and tail regions as well as the normal pericardium, yolk sac and swim bladder (**C-E**). The most severe malformations (*e.g.* EC – embryo coagulation, UHT – undeveloped head and tail, LS – lack of somites, POe – pericardial oedema, YSOe – yolk sac oedema, USB – uninflated swim bladder, CB – crooked body, TD – tail defect) that were observed in embryos and larvae treated with some test compounds (**F-J**) or pemetrexed (**K-O**). hpf – hours post-fertilization.
